# Potenzial und Limitationen von Schulimpfprogrammen zur Steigerung der HPV-Impfquoten in Deutschland

**DOI:** 10.1007/s00103-025-04029-1

**Published:** 2025-03-08

**Authors:** Anja Takla, Nora Schmid-Küpke, Ole Wichmann

**Affiliations:** 1 Fachgebiet Impfprävention, STIKO, Abteilung für Infektionsepidemiologie, Robert Koch-Institut, Berlin, Deutschland; 2 Robert Koch-Institut, Seestr. 10, 13353 Berlin, Deutschland

**Keywords:** Schulimpfungen, Implementation, HPV-Impfung, Evidenzbasiert, Impfsysteme, School vaccination, Implementation, HPV vaccination, Evidence-based, Immunization systems

## Abstract

Auch mehr als 15 Jahre nach der ersten Impfempfehlung der Ständigen Impfkommission (STIKO) gegen humane Papillomviren (HPV) sind die Impfquoten in Deutschland weiterhin niedrig. Als mögliche Maßnahme zur Steigerung der HPV-Impfquote in Deutschland wird häufig die flächendeckende Einführung von HPV-Schulimpfprogrammen gefordert, wie sie in anderen europäischen Ländern bestehen. Jedoch sollte jeder Implementation einer flächendeckenden Gesundheitsintervention eine Evidenzbewertung vorausgehen, die auch nationale Gegebenheiten berücksichtigt. In diesem Artikel wird ein Überblick über die bisher vorliegende Evidenz zum Effekt von Schulimpfprogrammen in Deutschland gegeben, die Ergebnisse werden eingeordnet. Entsprechende Evidenz ist bisher begrenzt und stammt aus 3 lokalen (Pilot‑)Programmen. Sie zeigt, dass etwa ein Drittel der Ungeimpften das Schulimpfangebot wahrnahm, zwei Drittel nahmen es nicht an. In keinem Programm in Deutschland wurden bislang Daten zu den Gründen für die Nichtannahme des Impfangebots erhoben, bzw. ob sich eine möglicherweise bestehende Skepsis auf den Impfort Schule oder die Impfung selbst bezieht. Darüber hinaus fehlen Aufwand- bzw. Kosten-Nutzen-Analysen von Schulimpfprogrammen, die für die Bewertung solcher in Deutschland neu zu etablierenden Strukturen wichtig wären. Auf Basis der bislang vorliegenden Evidenz erscheint es eher unwahrscheinlich, dass die Einführung eines flächendeckenden Schulimpfangebots die HPV-Impfquoten in Deutschland in relevantem Maße steigern kann. Schulimpfprogramme könnten ggf. aber – ergänzend zu anderen Maßnahmen – für spezifische Zielgruppen bzw. in bestimmten umschriebenen Regionen Teil eines strukturierten Impfsystems sein, welches – im Gegensatz zum bestehenden Impfsystem – in Deutschland sicherstellen könnte, dass allen in der Zielgruppe aktiv ein HPV-Impfangebot gemacht wird.

## Abstract (en)

Even more than 15 years after the first vaccination recommendation by the Standing Committee on Vaccination (STIKO) against human papillomaviruses (HPV), vaccination coverage in Germany remains low. As a possible measure to increase HPV vaccination coverage in Germany, the widespread introduction of HPV school vaccination programs, as they exist in other European countries, is often called for. However, any implementation of a widespread health intervention should be preceded by an evidence assessment that also takes national circumstances into account. This article provides an overview of the evidence available to date on the effect of school vaccination programs in Germany and discusses the results. Corresponding evidence is so far limited and comes from three local (pilot) programs. These show that about one-third of those not vaccinated took up the school vaccination offer, while two-thirds did not. No program in Germany has yet collected data on the reasons for not accepting the vaccination offer or whether any hesitancy that may exist relates to the place of vaccination (school) or the vaccination itself. Furthermore, there is a lack of cost–benefit analyses of school vaccination programs. This would be important for their evaluation due to the structures that would have to be newly established in Germany for this purpose. Based on the evidence available to date, it seems rather unlikely that the introduction of a comprehensive school vaccination program could significantly increase HPV vaccination rates in Germany. However, in addition to other measures, school vaccination programs could possibly be part of a structured vaccination system for specific target groups or in certain defined regions. In contrast to the existing vaccination system in Germany, such a structured vaccination system could ensure that all members of the target group are actively offered HPV vaccination.

## Einleitung

Jährlich erkranken etwa 8000 Menschen in Deutschland an Krebsarten, die durch humane Papillomviren (HPV) ausgelöst werden [1]. Häufigste Tumorlokalisation ist der Gebärmutterhals mit etwa 4600 jährlich neu auftretenden Erkrankungen. Die restlichen Tumorerkrankungen treten bei beiden Geschlechtern an den weiteren, für HPV-Infektionen typischen Lokalisationen des Analbereichs, Mund-Rachen-Raums sowie Genitalbereichs (Vagina, Vulva und Penis) auf.

Als mit Abstand wirksamste präventive Maßnahme steht seit 2006 eine Impfung gegen HPV zur Verfügung [1]. Aufgrund der weltweit hohen Anzahl an HPV-bedingten Krebserkrankungen und des hohen Präventionspotenzials der HPV-Impfung wurde von der Weltgesundheitsorganisation (WHO; [2]) und der EU-Kommission [3] das Ziel ausgegeben, dass Länder eine HPV-Impfquote von ≥ 90 % bei Mädchen (15-Jährige) und eine bedeutende Steigerung der Impfquoten bei den gleichaltrigen Jungen erreichen.

Bereits 2007 sprach in Deutschland die Ständige Impfkommission (STIKO) für alle 12- bis 17-jährigen Mädchen die Empfehlung zur HPV-Impfung aus [4], gefolgt 2014 von einer Herabsetzung des empfohlenen Impfalters auf 9–14 Jahre [5]. 2018 weitete die STIKO die Empfehlung auf alle Jungen aus [1]. Seither besteht eine geschlechtsneutrale HPV-Impfempfehlung für alle 9‑ bis 14-Jährigen (2 Impfdosen), mit der Möglichkeit zur Nachholimpfung bis zum 18. Geburtstag (3 Impfdosen). Die Umsetzung dieser STIKO-Empfehlung, wie auch die der anderen empfohlenen Impfungen, erfolgt in Deutschland aufgrund der Struktur des Gesundheitssystems fast ausschließlich durch niedergelassene Ärzt:innen [6].

Trotz der nun seit mehr als 15 Jahren bestehenden STIKO-Empfehlung lag die HPV-Impfquote 2023 bei 15-jährigen Mädchen in Deutschland lediglich bei 55 % (vollständige Impfserie [7]). Bei den gleichaltrigen Jungen war mit 34 % gerade ein Drittel der Altersgruppe vollständig gegen HPV geimpft [7].

Aufgrund dieser Impfquoten und des dadurch verpassten Potenzials, eine hohe Anzahl an Krebserkrankungen primär zu verhindern, besteht dringender Handlungsbedarf. Da viele Industrieländer höhere Impfquoten erreichen und in diesen oftmals auch Schulimpfprogramme implementiert sind, wird in Diskussionen zu möglichen Maßnahmen für eine Steigerung der HPV-Impfquoten in Deutschland oftmals die flächendeckende Einführung von Schulimpfprogrammen gefordert [8–10]. Schulimpfprogramme können definiert werden als das Angebot bzw. die Durchführung von Impfungen in der Schule, dem eine ausführliche Information bzw. Aufklärung und schriftliche Einwilligung der Sorgeberechtigten vorausgehen muss. Auch die im Mai 2020 publizierte S3-Leitlinie „Impfprävention HPV-assoziierter Neoplasien“ der Arbeitsgemeinschaft der Wissenschaftlichen Medizinischen Fachgesellschaften e. V. (AWMF) beinhaltet einen starken Konsens für die Empfehlung: „Eine Schulimpfung bezüglich HPV soll implementiert werden“ [11]. Im Juni 2021 benannte die Gesundheitsministerkonferenz aufsuchende Beratung/Erinnerung an Schulen (idealerweise durch den Öffentlichen Gesundheitsdienst), inklusive Impfangeboten vor Ort, als eine von mehreren empfohlenen Maßnahmen [12].

Nationale Impfempfehlungen sollten nach der WHO auf einer soliden Evidenzbewertung zu Impfstoff-Charakteristika, Krankheitslast, antizipierten Bevölkerungseffekten oder auch Aspekten wie Akzeptanz und Gerechtigkeit (engl. Equity) beruhen [13, 14]. Analog dazu sollte auch die Implementierung von neuen flächendeckenden Gesundheitsinterventionen evidenzbasiert erfolgen. Ziel dieses Artikels ist es daher, die bisher vorliegende Evidenz zu Effekten von Schulimpfprogrammen in Deutschland zusammenzutragen sowie die Ergebnisse einzuordnen.

## Evidenz zum Effekt von Schulimpfprogrammen in Deutschland

Die bisher vorliegende Evidenz zum Effekt von Schulimpfprogrammen in Deutschland ist begrenzt und stammt aus 3 lokalen (Pilot‑)Programmen [15–17]. Neben dem Zielgruppenalter unterscheiden sich die Programme auch in weiteren Parametern (Tab. 1).

**Tab. 1:** Auflistung ausgewählter Parameter der 3 HPV-Schulimpfprogramme in Deutschland (Stand 2024; [15–17, 24])

	Landkreis Bergstraße (Hessen)	Stadtkreis Leipzig (Sachsen)	Stadtkreis Bremen (Bremen)
Setting	Ländlich	Städtisch	Städtisch
Beginn	2015/2016	2019/2020	2013/2014
Zielgruppe	4. Klassen (9- bis 10-Jährige) Mädchen (bis Pandemiebeginn) Mädchen und Jungen (seit 2023/2024)	4. und 5. Klassen (9- bis 11-Jährige) Mädchen und Jungen	8. Klassen (13- bis 14-Jährige) Mädchen (bis 2018/2019) Mädchen und Jungen (seit 2022/2023)
Initiator(en)	Gesundheitsnetz Rhein-Neckar e. V., preventa Stiftung	HPV-Schulimpfprojekt (Dr. Hösemann)	Gesundheitsamt Bremen (ÖGD)
Durchführung durch	Freiwillige (niedergelassene Ärzt:innen unterschiedlicher Fachrichtungen)	Freiwillige (niedergelassene Ärzt:innen unterschiedlicher Fachrichtungen)	Gesundheitsamt Bremen

### Schulimpfprogramm im Landkreis Bergstraße

Das Modellprojekt „Freiwillige HPV-Schulimpfung“ wurde im Schuljahr 2015/2016 initiiert und begann im Landkreis (LK) Bergstraße mit 6 Pilotschulen [15]. Zielgruppe waren Mädchen der 4. Klassenstufe (9- bis 10-Jährige). Über die HPV-Impfung und den Ablauf wurde während eines Elternabends durch den Projektträger und eine/n Impfärzt:in aufgeklärt. Alternativ zum Impfort Schule konnte die Impfung auch in der Praxis stattfinden. Bis zu den Schuljahren 2018/2019 und 2019/2020 stieg die Anzahl der teilnehmenden Schulen auf 19 von insgesamt 52 Schulen mit 4. Klassen im LK Bergstraße. Diese Schulen umfassten 49 % (528/1073) bzw. 48 % (539/1112) aller Viertklässlerinnen im LK. Damit wurde theoretisch die Hälfte aller Mädchen bzw. deren Eltern im LK über das HPV-Schulimpfangebot informiert [18]. Vor Kurzem wurde das Modellprojekt wieder aufgenommen [19].

Bisher liegt eine Evaluation zum Effekt des Schulimpfprogramms aus dem ersten Projektjahr 2015/2016 vor [20]. Diese umfasste die Befragung von 202 Eltern mit Kindern an den 6 teilnehmenden Schulen. 58 % von diesen waren nach dem Elternabend an der HPV-Impfung interessiert, davon mit 55 % etwas mehr an einer Impfung in der Arztpraxis als in der Schule (45 %). Die Hälfte (*n* = 62) der interessierten Eltern füllte später einen Kurzfragebogen aus. 77 % (*n* = 48) gaben die Impfung ihrer Tochter an, davon 56 % in der Praxis und 44 % in der Schule.

Um den Effekt des Schulimpfangebots auf die HPV-Impfquote der Mädchen im LK Bergstraße zu analysieren, wurden Daten der kassenärztlichen Vereinigungen (KVen) genutzt [18]. Dies war möglich, da mittlerweile knapp 50 % aller Mädchen im LK durch das Schulimpfangebot erreicht worden waren. Aus den Ergebnissen der Evaluation wurde geschlussfolgert, dass das Schulimpfangebot im LK Bergstraße im Vergleich zu anderen hessischen LK zu mehr vervollständigten Impfserien innerhalb von 12 Monaten geführt hat. Darüber hinaus konnte in den Daten eine frühzeitigere Impfung der 10-jährigen Mädchen im Vergleich zu den anderen LK bzw. dem hessischen Durchschnitt beobachtet werden. Der Unterschied betrug in dieser Altersgruppe bis zu 10 Prozentpunkte für die vollständige und bis zu 23 Prozentpunkte für die angefangene Impfserie. Im weiteren Altersverlauf konnte jedoch keine Erhöhung der Impfquoten im Vergleich zu den anderen LK ohne Schulimpfangebot gesehen werden. Um diesen Aspekt abschließend zu beurteilen, müsste der Beobachtungszeitraum für die Evaluation jedoch ausgeweitet werden. Dennoch geben die Ergebnisse Hinweise darauf, dass unsichere oder impfskeptische Eltern durch das Schulimpfprogramm vermutlich nicht erreicht werden und es stattdessen als niederschwelliger Zugang für bereits impfwillige Eltern fungieren könnte (die sonst die Praxis gewählt hätten). Aus einem in der Evaluation vorgestellten hypothetischen Szenario war es am wahrscheinlichsten, dass die Impfquote unter den Viertklässlerinnen der am Impfprogramm teilnehmenden Schulen bei ~40 % lag. Ob diese Annahme zutrifft und ob diese HPV-Impfungen in der Schule oder – wie ebenfalls angeboten – in der Praxis stattgefunden haben, ist unklar.

### Schulimpfprogramm im Stadtkreis Leipzig

Das HPV-Schulimpfprojekt im Stadtkreis (SK) Leipzig wurde 2018 als Initiative von Ärzt:innen, Apotheker:innen und Gesundheitswirt:innen gegründet. Projektziel ist die „Vorbereitung, Aufklärung der Eltern und Durchführung der HPV-Impfung direkt in Schulen, um alle Schüler*innen zu erreichen und zu impfen“ [16]. Information und Aufklärung der Eltern erfolgt von freiwilligen Impfärzt:innen während Elternabenden, das Impfangebot findet im schulischen Umfeld statt. Zielgruppe sind Mädchen und Jungen in 4./5. Klassen (9- bis 11-Jährige).

Bisher wurden 2 programminterne Evaluationen aus den Schuljahren 2020/2021 [21] und 2023/2024 [22] veröffentlicht. Im Schuljahr 2020/2021 betrug der Anteil der Jungen und Mädchen mit ≥ 1 HPV-Impfung im Rahmen des Schulimpfprogramms 34,2 % und mit vollständiger HPV-Impfserie 28,3 % (*N* = 512). Vor dem Schulimpfangebot waren bereits 8,8 % der Mädchen HPV-geimpft. Für das Schuljahr 2023/2024 lagen bisher nur Daten für ≥ 1 HPV-Impfung innerhalb des Schulimpfprogramms vor – hier lag der Anteil geimpfter Jungen und Mädchen bei 29,1 % (*N* = 505). Die erste Evaluation 2020/2021 beinhaltete zusätzlich eine Elternbefragung zur Impfbereitschaft. Die Teilnahmequote betrug 47 % (*n* = 241), von diesen beurteilten 87 % die Möglichkeit einer Schulimpfung positiv, 74 % wünschten sich weitere Informationen zur Impfung an Schulen und 21 % gaben an, dass das Projekt einen Einfluss auf die Impfbereitschaft hatte.

### Schulimpfprogramm im Stadtkreis Bremen

Das HPV-Schulimpfprogramm in Bremen ist eine Initiative des Gesundheitsamtes (GA) Bremen und wurde erstmalig im Schuljahr 2013/2014 durchgeführt [23]. Zielgruppe sind Mädchen aller 8. Klassen (13- bis 14-Jährige) im SK Bremen. Hintergrund für die Wahl der 8. Klassenstufe war, dass die Schulimpfungen nicht in Konkurrenz zur Impfung in Praxen stehen sollten. Zudem sollten die Schulimpfungen als Angebot zur Schließung letzter Impflücken kurz vor Ende des empfohlenen Impfzeitraums von 9–14 Jahren fungieren. Zwischen 2020/2021 und 2021/2022 musste das Programm aufgrund der COVID-19-Pandemie ausgesetzt werden und wurde dann ab dem Schuljahr 2022/2023 – entsprechend der STIKO-Empfehlung – neu auch auf alle Schüler der 8. Klassen ausgedehnt. Die Verteilung von Infomaterial zur HPV-Impfung für die Sorgeberechtigten erfolgte jeweils über die Klassenlehrer:innen. Neben Infomaterial wurde auch ein Fragebogen verteilt, der den aktuellen HPV-Impfstatus und die Einwilligung bzw. Nichtannahme des schulischen HPV-Impfangebots erfasste. Der Fragebogen enthielt dazu das Angebot für eine individuelle telefonische Impfberatung durch das GA Bremen. Die Schulimpfungen wurden jeweils vor Ort durch das Impfteam des GA durchgeführt und dokumentiert. Eine detaillierte Beschreibung des organisatorischen Aufwandes und der Durchführung wurde vom GA Bremen publiziert (siehe Beitrag von Renken et al. in diesem Themenheft).

Nach einer ersten Publikation von Ergebnissen aus dem Schuljahr 2018/2019 [24] erfolgte eine umfassende Evaluation des Schulimpfprogramms über den Zeitraum 2015/2016 bis 2022/2023 durch das GA Bremen und das Robert Koch-Institut (RKI; [23]). Neben der Evaluation der Inanspruchnahme nach Geschlecht und Schuljahr (prä- vs. postpandemisch) wurden auch mögliche Unterschiede in der Inanspruchnahme des Impfangebots nach Schulsozialindex untersucht. Dieser bildet die sozioökonomische Zusammensetzung der Schülerschaft einer Schule ab. Das Bremer Schulamt kategorisiert jede Schule entsprechend einem Index von 1 (wenig Belastung) bis 5 (hohe Belastung). In den Index fließen 7 Indikatoren ein, die sich auf individuelle Schüler:innendaten u. a. zu Armut, Lebensumwelt und Migration/Integration stützen [25, 26].

Die Evaluation bezog Daten aus 5 Schuljahren bzw. von 10946 Schülerinnen und 2404 Schülern in 1140 Klassen aus 56 Schulen ein. Die Rücklaufquote der Fragebögen lag für die 4 präpandemischen Schuljahre bis 2018/2019 zwischen 76 % und 84 %. Nach pandemiebedingter Pause nahm die Rücklaufquote um 28 Prozentpunkte auf 56 % ab. Über die Jahre stieg der Anteil von Mädchen, die bereits vor dem Schulimpfangebot in einer Praxis geimpft wurden, kontinuierlich an (2015/2016: 18 %; 2022/2023: 46 %). Unter Kindern mit der geringsten Belastung gemäß Schulsozialindex lag dieser Anteil mit 40 % signifikant höher als in den anderen Kategorien (24–26 %).

Das Bremer Schulimpfangebot wurde von 26–39 % (≥ 1 HPV-Impfung) bzw. 22–28 % (vollständige Impfserie) der ungeimpften 13- bis 14-Jährigen in Anspruch genommen. Die Rücklaufquote der Fragebögen war in der Gruppe mit der höchsten Belastung (Schulsozialindex) im Vergleich zu den anderen Kategorien niedriger, die Bereitschaft zur Schulimpfung aber etwas höher. Insgesamt konnte die HPV-Impfquote im SK Bremen für das Jahr 2019 durch das Schulimpfprogramm von 37,1 % (15-jährige Mädchen, vollständige Impfserie) um etwa 15 Prozentpunkte auf 51,9 % gesteigert werden [27]. Vergleichbare Steigerungen finden sich auch für die Jahre 2017 und 2018. Darüber hinaus stützt v. a. für die Jahre 2017 bis 2019 eine vermehrte Anzahl von HPV-Erstimpfungen in Praxen (Daten KV-Impfsurveillance) im zeitlichen Zusammenhang mit dem Schulimpfangebot im SK Bremen einzelne Berichte von niedergelassenen Kinderärzt:innen über Eltern, die mit Infomaterial aus dem Schulimpfprogramm und einem HPV-Impfwunsch in der Praxis vorstellig wurden [27]. Im SK Bremerhaven ohne HPV-Schulimpfprogramm lässt sich in den KV-Daten keine vergleichbare Steigerung beobachten.

## Zu welchen Fragestellungen im Kontext HPV-Schulimpfprogramme fehlt Evidenz?

Um das Potenzial von Schulimpfprogrammen zur Steigerung der HPV-Impfquoten in Deutschland beurteilen und fundierte gesundheitspolitische Entscheidungen treffen zu können, mangelt es nach wie vor an Evidenz zu den folgenden Fragestellungen.

### Elterliche Impfortpräferenz, HPV-Impfeinstellung und Zugangsgerechtigkeit (Equity)

Um zu verstehen, welche Eltern ihre Kinder im Rahmen eines HPV-Schulimpfprogramms impfen lassen, sollten elterliche Präferenzen für HPV-Impforte und die Gründe für die jeweilige Wahl erhoben werden. So lassen sich strukturelle Barrieren bei Impfangeboten erkennen und ausräumen. Zudem kann eruiert werden, ob bestimmte Elterngruppen über ein HPV-Schulimpfangebot erstmalig und nur über die Schule erreicht werden. Wichtig ist, auch den Grad an Impfskepsis der Eltern zu ermitteln [28]. Dieser gibt Aufschluss darüber, ob eher impfskeptische Eltern bzw. Gruppen ohne/mit limitiertem Zugang zum etablierten Praxissystem erreicht werden. Die Erhebung sozialer Determinanten kann beispielsweise die Frage beantworten, ob vulnerable Gruppen erreicht werden. Nicht zuletzt sollten weitere (z. B. sprachliche) Hürden betrachtet werden, die einen fairen Zugang zu Impfinformationen und -angeboten behindern könnten. Eine systematische Untersuchung kann klären, inwiefern ein Schulimpfprogramm zu mehr Equity beitragen kann [29].

### Aufwand- bzw. Kosten-Nutzen-Analysen von HPV-Schulimpfprogrammen

Die Evaluation des Potenzials von HPV-Schulimpfprogrammen zur Steigerung der HPV-Impfquote sollte auch eine Analyse des Aufwandes bzw. der Kosten vs. des Nutzens des Programms beinhalten. Im Aufwand werden z. B. Personenanzahl und -stunden für Vorbereitung, Durchführung und Nachbereitung des Schulimpfangebots erfasst. Eine ausführliche Beschreibung der notwendigen Tätigkeiten und des Personalaufwandes wurde erstmals vom GA Bremen veröffentlicht (siehe Beitrag von Renken et al. in diesem Themenheft). Diese Aufwandserfassung trifft auch auf Schulimpfprogramme zu, die durch freiwillige Impfärzt:innen durchgeführt werden: Hier muss berücksichtigt werden, dass diese an Schulimpftagen ggf. Arbeitsausfallzeiten in ihren Praxen haben, sodass sich ihr Engagement indirekt auf die Regelversorgung von anderen Patient:innen auswirken kann. Demgegenüber stehen die durch die Impfung direkt und indirekt vermiedenen Krankheitskosten sowie der gewonnene Nutzen im Erhalt der Gesundheit und Lebensqualität durch die Vermeidung von Tumorerkrankungen und Genitalwarzen. Diese Aufwand‑/Kosten-Nutzen-Analysen können auch für spezielle Subgruppen durchgeführt werden, z. B. bezogen auf Regionen oder Schulen mit einem hohen Anteil an Kindern mit erschwertem Zugang zum etablierten Praxissystem, in Regionen ohne ausreichende kinderärztliche Versorgung oder für Schulimpfprogramme speziell im ländlichen Raum. Um Aufwand/Kosten-Nutzen einzuordnen, könnte das HPV-Schulimpfprogramm mit anderen Maßnahmen zur Steigerung von HPV-Impfquoten verglichen werden [30].

## Zusammenfassung und Einordnung der Evidenz

Bei 2 der 3 (Pilot‑)Schulimpfprogrammen stammt die vorliegende Evidenz zum Effekt auf die HPV-Impfquote direkt aus programminternen Daten (SK Leipzig, SK Bremen) und erlaubt erste Einschätzungen zum Potenzial dieser Intervention in Deutschland. Obwohl sich das Alter der Zielgruppen mit 9–11 Jahren (SK Leipzig) und 13–14 Jahren (SK Bremen) deutlich unterschied, wurde das HPV-Schulimpfangebot in beiden Programmen recht konstant von etwa einem Drittel (≥ 1 Impfung) bzw. einem Viertel (vollständige Impfserie) der ungeimpften Schüler:innen in Anspruch genommen. Dieser Anteil war gleichbleibend, auch bei unterschiedlich hohem Anteil von bereits vor dem Schulimpfangebot geimpften Mädchen (SK Leipzig: 8,8 % bzw. SK Bremen: 18–46 %). Für das dritte Schulimpfprogramm (LK Bergstraße) liegt bisher keine mehrjährige Evaluation aus programminternen Daten vor. Die indirekte Effektevaluation auf LK-Ebene ergab, dass das Schulimpfprogramm in der Zielgruppe der Viertklässlerinnen zu einer HPV-Impfquote von wahrscheinlich ~40 % geführt hat. In der publizierten programminternen Befragung aus dem Jahr 2015/2016 gaben 55 % der Eltern als Impfort die Praxis an, 45 % die Schule. Ob dies auch auf die späteren Jahre des Schulimpfprogramms zutrifft, ist unbekannt.

Wie die Gruppe der Eltern charakterisiert ist, die das HPV-Schulimpfangebot in Anspruch nehmen, ist aufgrund fehlender Daten bisher unklar. Es ist möglich, dass durch das Schulimpfangebot Eltern angesprochen werden, die *neu* und *nur* über die Schule erreicht werden. Dies könnten Familien sein, die keinen oder nur einen begrenzten Zugang zum etablierten Praxissystem haben, z. B. aufgrund einer generell schwachen Infrastruktur des Gesundheitssystems vor Ort oder anderer Charakteristika wie Bildungsstand oder Sprache. Eine andere Möglichkeit ist, dass diejenigen Eltern das Schulimpfangebot in Anspruch nehmen, die der HPV-Impfung und dem angebotenen Impfort (bereits) positiv gegenüberstehen und die Schulimpfung als niedrigschwelligen Zugang im Vergleich zur (kinderärztlichen) Praxis betrachten. In Bremen ist dies trotz der vom GA bewusst gewählten höheren Klassenstufe nicht auszuschließen: Auswertungen aus der KV-Impfsurveillance zeigten, dass im SK Bremen 39–52 % aller geimpften Mädchen im empfohlenen Impfalter ihre erste HPV-Impfung im Alter von 13 oder 14 Jahren in Praxen erhielten [27]. Damit fungiert das Schulimpfangebot ungewollt als zeitgleiche Impfalternative zur Praxis – trotz Intention des GA, ein den Praxen zeitlich nachgeordnetes HPV-Impfangebot zu etablieren. Gleichzeitig legen die KV-Daten aus dem SK Bremen für einzelne Jahre einen möglichen indirekten Effekt durch Schulimpfangebote auf die HPV-Impfquote nahe. Das Schulimpfangebot fungiert für einige Eltern wahrscheinlich als Erinnerung an die HPV-Impfung, die jedoch eine Impfung bei einem/einer niedergelassenen Pädiater:in (mit möglicherweise weiterer Impfberatung) bevorzugen. Schließlich fehlt bisher für den deutschen Kontext Evidenz zum Aufwand- bzw. Kosten-Nutzen-Verhältnis von Schulimpfprogrammen – auch im Vergleich zu anderen impfquotensteigernden Maßnahmen.

## Blick über den Tellerrand: HPV-Impfquote und Impfort in anderen europäischen Ländern

Deutschland rangiert mit einer HPV-Impfquote bei 15-jährigen Mädchen von 55 % (vollständige Impfserie, 2023) im europäischen Vergleich eher im unteren Drittel (Tab. 2). Ein genauerer Blick auf diejenigen Länder in Europa, die seit Jahren hohe HPV-Impfquoten erreichen, ist daher naheliegend. Hierzu wurde für die Länder Europas Tab. 2 auf Basis von publizierten Daten zu HPV-Impfquoten und Impfort erstellt [31, 39, 40]. Ein Teil dieser Länder bietet die HPV-Impfung im Rahmen von Schulimpfprogrammen an, so z. B. Norwegen, Schweden, Spanien oder Ungarn. Andere Länder mit hohen HPV-Impfquoten wie Portugal, Dänemark, Malta oder Litauen bieten die HPV-Impfungen ausschließlich in Praxen und/oder Gesundheitszentren an.

**Tab. 2:** HPV-Impfquote für 15-jährige Mädchen (vollständige Impfserie, 2023; [39]) und hauptsächlicher HPV-Impfort in verschiedenen europäischen Ländern. Die Informationen zum Impfort basieren auf Angaben aus dem PERCH (PartnERship to Contrast HPV) Report Work Package 4 ([40]; Länder in der Tabelle gekennzeichnet mit *, Datenstand: 2023), ergänzt durch Angaben aus Nguyen-Huu et al. ([31]; Datenstand: 2010–2017**)**

Land	HPV-Impfquote (in %)	Impfort		
		Schulen	Gesundheitszentren	Praxen
Island	96	x	x	–
Norwegen*	93	x	–	–
Portugal	91	–	x	x
Spanien*	85	x	x	–
Schweden*	85	x	–	–
Dänemark	83	–	–	x
Malta	82	–	x	–
Finnland	76	x	–	–
Litauen*	76	–	x	x
Ungarn*	76	x	–	–
Irland	75	x	x	–
Belgien*	72	x	–	–
Tschechien*	71	–	x	x
Schweiz	70	x	x	x
Zypern	67	x	–	x
UK	65	x	x	x
Niederlande	65	–	x	x
Italien*	64	–	x	–
Deutschland*	55	–	–	x
Österreich	53	x	x	x
Slowenien*	52	x	–	–
Lettland	46	–	x	x
Frankreich*	45	–	–	x
Estland*	43	x	–	–
Luxemburg	43	–	–	x
Bulgarien	7	–	x	x

## Theoretische Überlegungen zu Einflussfaktoren auf die HPV-Impfquote

**Abb. 1:**
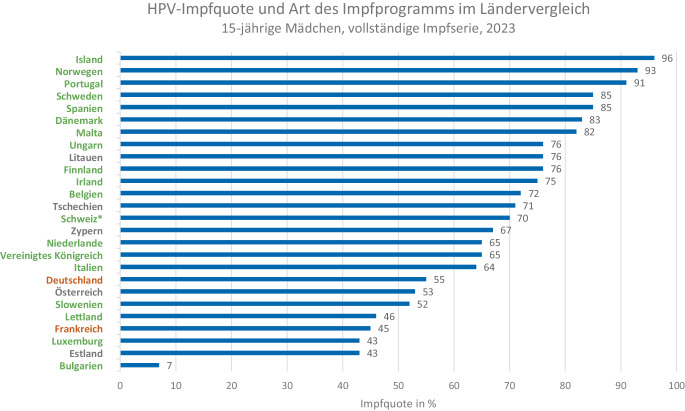
HPV-Impfquoten [39] und Art des Impfprogrammes in verschiedenen europäischen Ländern (basierend auf Nguyen-Huu et al. [31]). Länder mit strukturiertem Impfsystem sind in grün, Länder mit einem „opportunistischen“ Impfsystem in orange und Länder ohne Angaben in grau gekennzeichnet. Länder mit einem teils strukturierten System sind mit * versehen. Quelle: eigene Abbildung

**Abb. 2:**
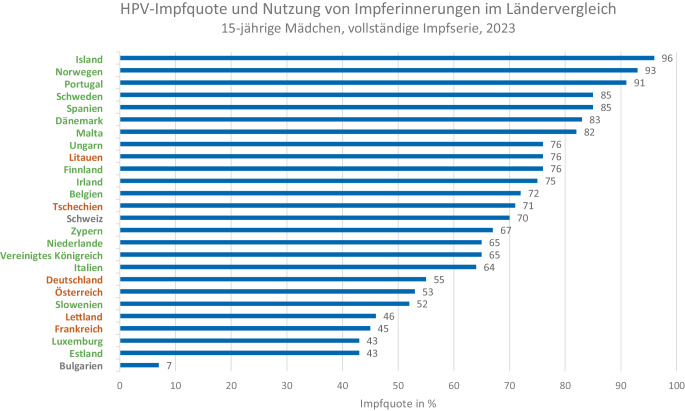
HPV-Impfquote [39] und Nutzung von Impferinnerungen in verschiedenen europäischen Ländern (basierend auf Nguyen-Huu et al. [31]). Länder, die Impferinnerungen nutzen, sind in grün gekennzeichnet, Länder ohne Impferinnerung in orange und Länder ohne Angaben in grau. Quelle: eigene Abbildung

Aufgrund der in Tab. 2 dargestellten Information kann argumentiert werden, dass der Ort, an dem die Impfung angeboten und durchgeführt wird, nicht *per se* der Grund für hohe oder niedrige HPV-Impfquoten ist. Eine andere Möglichkeit, Länder auf europäischer Ebene in Bezug auf ihre HPV-Impfquoten zu vergleichen, wäre die Unterscheidung nach Vorliegen eines strukturierten oder „opportunistischen“ Impfprogramms [31]. Während in strukturierten Impfprogrammen systematisch allen Personen innerhalb der Zielgruppe aktiv ein Impfangebot gemacht wird, basiert ein „opportunistisches“ Programm darauf, dass die Person den Impfort aufsucht (auch aus anderen Gründen als einer gewünschten Impfung) und dort entweder selbst nach der Impfung fragt oder ihr dort ein Impfangebot unterbreitet wird. Abb. 1 zeigt die HPV-Impfquote (15-jährige Mädchen, vollständige Impfserie, 2023) und die Art des Impfprogramms (strukturiert versus „opportunistisch“) für die einzelnen europäischen Länder. Abb. 2 stellt dar, welche Länder Impferinnerungen für nicht wahrgenommene HPV-Impfungen nutzen.

Dennoch erreichen auch mit einem strukturierten Impfprogramm nur wenige Länder in Europa Impfquoten ≥ 90 %. Ein Grund hierfür ist, dass neben dem Zugang zur HPV-Impfung auch der Grad an Impfskepsis und institutionellem Vertrauen in der Bevölkerung eine relevante Rolle für die erreichbare Impfquote spielt [32]. Immer wieder zeigen Studien außerdem den großen Einfluss der ärztlichen Empfehlung auf die Impfentscheidung von Patient:innen [33]. Die Impfeinstellung des medizinischen Personals sowie dessen kommunikative Fähigkeiten im Gespräch mit Patient:innen sind daher ebenso bedeutsam. In einer Studie des europäischen JitsuVAX-Projektes [34] wurde ärztliches Personal in Frankreich (*N* = 1213), Finnland (*N* = 375), Portugal (*N* = 557) und Deutschland (*N* = 603) zur „wahrgenommenen Impfstoffsicherheit“, zum „Vertrauen in Gesundheitsbehörden“ und zum „Commitment zur Impfung“ befragt [35]. Während Portugal in allen 3 Bereichen hohe Werte zeigte, waren die Werte in Deutschland und Frankreich im Vergleich zu Portugal und auch Finnland v. a. für „Vertrauen in Gesundheitsbehörden“ deutlich geringer. Auch die „wahrgenommene Impfstoffsicherheit“ war in Deutschland und Frankreich geringer ausgeprägt als in Portugal und Finnland. Beim Thema „Commitment zur Impfung“ waren die Werte für Deutschland im Vergleich zu Portugal geringer, aber höher als in Frankreich und Finnland. Gemeinsam mit der Art des Impfprogramms („opportunistisch“ vs. strukturiert) bieten die Ergebnisse der Studie einen Erklärungsansatz dafür, warum Portugal (mit strukturiertem Impfsystem und hohem Vertrauen in Gesundheitsbehörden) zu den Ländern mit der höchsten HPV-Impfquote gehört, während Deutschland und Frankreich sich (mit opportunistischem Impfsystem und niedrigem Vertrauen) eher im unteren Drittel bewegen.

Impfungerechtigkeit („immunization inequity“) ist definiert als vermeidbare Impfquotenunterschiede, die benachteiligte Personen oder Gruppen einem erhöhten Risiko einer impfpräventablen Erkrankung aussetzen [29]. Die HPV-Impfquotendifferenz von bis zu 54 bzw. 63 Prozentpunkten (15-jährige Mädchen bzw. Jungen, vollständige Impfserie) zwischen den einzelnen Landkreisen stellt einen deutlichen Indikator für eine in Deutschland bestehende Ungerechtigkeit dar [7, 29]. Im Sinne des Equity-Gedankens muss jedoch sichergestellt sein, dass allen Kindern und Jugendlichen im empfohlenen HPV-Impfalter – unabhängig von Faktoren wie der aktiven Arzt-Patient-Beziehung oder dem Wohnort – die HPV-Impfung angeboten wird. Zur Reduzierung dieser Ungerechtigkeit sollte ein praxisunabhängiges Einladungs- und Erinnerungssystem genutzt werden, das die Zielgruppe z. B. aufgrund der bestehenden Schulpflicht über die Schule oder aufgrund der bestehenden Krankenversicherungspflicht über die individuelle Krankenkasse oder über eine Ausweitung des verbindlichen Einladewesens der Früherkennungsuntersuchungen U1–U9 durch die Bundesländer [36] auf die J1 (12- bis 14-Jährige; bzw. die „neue U10“ [37]) erreicht.

Gleichzeitig ist es für hohe HPV-Impfquoten unerlässlich, dass die in Deutschland vorhandene HPV-Impfskepsis adressiert wird. Dass bei einem Teil der Eltern eine Zögerlichkeit gegenüber der HPV-Impfung vorliegt, darauf weisen bisher unveröffentlichte Daten einer bundesweiten Repräsentativbefragung von 1500 Eltern mit Kindern im Alter von 9–14 Jahren in Deutschland im Herbst 2023 [38] hin: Hier zeigten ca. 35 % der befragten Eltern ungeimpfter Kinder einen unterschiedlichen Grad an HPV-Impfskepsis. Wichtig wäre zu untersuchen, welche Orte impfzögerliche Eltern am ehesten für eine HPV-Impfberatung (z. B. Informationsveranstaltung/Elternabend in der Schule oder Beratungsgespräch in der Praxis) und ggf. Impfung (z. B. Schule oder Praxis) präferieren würden. Dabei sollte auch berücksichtigt werden, wie gut sich kommunikative Techniken bei der Impfberatung an den verschiedenen Impforten umsetzen lassen. Diese Erkenntnisse sollten in die evidenzbasierte Entscheidung miteinfließen, welche Strategie (für ggf. welche Subgruppen) zur Steigerung der HPV-Impfquoten in Deutschland am effektivsten sein könnte.

## Fazit

Entscheidungen in Bezug auf die Implementierung von Maßnahmen zur Impfquotensteigerung sollten evidenzbasiert erfolgen. Dabei muss jedoch berücksichtigt werden, dass Ergebnisse aus anderen Ländern oder mit anderen Impfstoffen ggf. nur eingeschränkt übertragbar sind. Daher ist es wichtig, kontextspezifische Evaluationen durchzuführen und diese, soweit vorhanden, als Grundlage für Entscheidungen zu Implementationsstrategien zu nutzen.

Die bisher vorliegende Evidenz aus Deutschland zeigt, dass Schulimpfprogramme in Deutschland die HPV-Impfquoten steigern können. Sie stellen ein strukturiertes Impfangebot dar, wodurch alle Kinder bzw. Jugendlichen ein HPV-Impfangebot erhalten – einschließlich derjenigen ohne oder mit nicht genutzter kinderärztlicher Versorgung und bei adäquater Adressierung von möglichen sprachlichen Barrieren. Die bisher aus Deutschland vorliegenden Evaluationsergebnisse zeigen, dass bis zu einem Drittel der Ungeimpften das Impfangebot in Schulen annehmen. Im SK Bremen führte das Schulimpfprogramm über mehrere Jahre zu einer jährlichen Impfquotensteigerung von ~15 Prozentpunkten bei 15-jährigen Mädchen (vollständige Impfserie).

Gleichzeitig ist es aufgrund der bisher vorliegenden Evidenz eher unwahrscheinlich, dass die Einführung von flächendeckenden Schulimpfprogrammen in Deutschland zu HPV-Impfquoten von ≥ 90 % und damit zum Erreichen der Ziele von WHO bzw. EU-Kommission führen wird. Etwa zwei Drittel der Ungeimpften nahmen über den beobachteten Zeitraum und unabhängig von Geschlecht und Schulsozialindex das Schulimpfangebot nicht an. Im SK Bremen lag die HPV-Impfquote trotz flächendeckenden Schulimpfprogramms unter 15-jährigen Mädchen bei etwas über 50 % (vollständige Impfserie).

Die Höhe der erreichten HPV-Impfquote in einem Land setzt sich aus dem Angebot und Zugang zur HPV-Impfung sowie dem Grad an HPV-Impfskepsis und institutionellem Vertrauen zusammen. Grundlage für einen ubiquitären (und damit gerechten) Zugang zur HPV-Impfung sollte ein strukturiertes Impfsystem sein, welches sicherstellt, dass allen Personen in der Zielgruppe *aktiv* ein Impfangebot gemacht wird. Dies ist in Deutschland bisher nicht der Fall, da ein HPV-Impfangebot im Regelfall von einer aktiven Arzt-Patient-Beziehung mit Praxisbesuchen abhängt. Ein praxisunabhängiges Einladungs- und Erinnerungssystem, welches die Zielgruppe z. B. aufgrund der bestehenden Schulpflicht über die Schule, aufgrund der bestehenden Krankenversicherungspflicht über die individuelle Krankenkasse oder über eine Ausweitung des verbindlichen Einladewesens der Früherkennungsuntersuchungen der Länder auf die J1 bzw. „neue U10“ [37] erreicht, könnte dies sicherstellen. Ein anderer, relevanter Faktor für die Ausgestaltung eines strukturierten HPV-Impfangebots in Deutschland ist die Kenntnis darüber, welche elterlichen Präferenzen für mögliche (HPV-)Impforte wie Schule, ärztliche Praxis, GA oder z. B. Impfzentren bestehen. Darüber hinaus bedarf es an Evidenz, ob, und wenn ja, wie und über welchen bevorzugten Impfort impfskeptische Eltern am besten für eine HPV-Impfaufklärung und -beratung erreicht werden können. Abhängig von diesen Ergebnissen und lokalen Gegebenheiten könnten Schulimpfprogramme möglicherweise für spezifische Zielgruppen bzw. in bestimmten Regionen Impfungerechtigkeiten mittels eines aktiven Impfangebots adressieren.

Unter Einbeziehung all dieser Faktoren bedarf es schließlich Aufwand- bzw. Kosten-Nutzen-Analysen für die verschiedenen Möglichkeiten von strukturierten HPV-Impfangeboten und deren Effekten auf die HPV-Impfquoten, um die knappen Ressourcen im Gesundheitsbereich möglichst effizient einzusetzen. Eine Herausforderung ist, dass die hierfür notwendigen gesundheitspolitischen Maßnahmen eine Mischung aus Bundes‑, Länder- und kommunaler Zuständigkeit darstellen. Bei einer Festlegung auf eine gemeinsame Strategie sind daher eine gute Absprache und Koordination mit den verschiedenen Akteuren sowie – je nach Strategie – die Einbeziehung der Krankenkassen und niedergelassenen Ärzteschaft notwendig. In jedem Fall sind nach den Erfahrungen der letzten 15 Jahre und der zusammengetragenen Evidenz strukturelle Änderungen im deutschen Impfsystem notwendig, um das erwiesene Präventionspotenzial der HPV-Impfung allen Personen der Zielgruppe zugänglich zu machen.
